# Brain damage in methylmalonic aciduria: 2-methylcitrate induces cerebral ammonium accumulation and apoptosis in 3D organotypic brain cell cultures

**DOI:** 10.1186/1750-1172-8-4

**Published:** 2013-01-08

**Authors:** Paris Jafari, Olivier Braissant, Petra Zavadakova, Hugues Henry, Luisa Bonafé, Diana Ballhausen

**Affiliations:** 1 Inborn Errors of Metabolism, Molecular Pediatrics, Lausanne University Hospital, 1011 Lausanne, Switzerland; 2 Inborn Errors of Metabolism, Biomedicine, Lausanne University Hospital, 1011 Lausanne, Switzerland

**Keywords:** Methylmalonic aciduria, Methylmalonate, 2-methylcitrate, Propionate, Hyperammonemia, Apoptosis, Brain damage, Neurotoxicity

## Abstract

**Background:**

Methylmalonic aciduria is an inborn error of metabolism characterized by accumulation of methylmalonate (MMA), propionate and 2-methylcitrate (2-MCA) in body fluids. Early diagnosis and current treatment strategies aimed at limiting the production of these metabolites are only partially effective in preventing neurological damage.

**Methods:**

To explore the metabolic consequences of methylmalonic aciduria on the brain, we used 3D organotypic brain cell cultures from rat embryos. We challenged the cultures at two different developmental stages with 1 mM MMA, propionate or 2-MCA applied 6 times every 12 h. In a dose–response experiment cultures were challenged with 0.01, 0.1, 0.33 and 1 mM 2-MCA. Immunohistochemical staining for different brain cell markers were used to assess cell viability, morphology and differentiation. Significant changes were validated by western blot analysis. Biochemical markers were analyzed in culture media. Apoptosis was studied by immunofluorescence staining and western blots for activated caspase-3.

**Results:**

Among the three metabolites tested, 2-MCA consistently produced the most pronounced effects. Exposure to 2-MCA caused morphological changes in neuronal and glial cells already at 0.01 mM. At the biochemical level the most striking result was a significant ammonium increase in culture media with a concomitant glutamine decrease. Dose–response studies showed significant and parallel changes of ammonium and glutamine starting from 0.1 mM 2-MCA. An increased apoptosis rate was observed by activation of caspase-3 after exposure to at least 0.1 mM 2-MCA.

**Conclusion:**

Surprisingly, 2-MCA, and not MMA, seems to be the most toxic metabolite in our *in vitro* model leading to delayed axonal growth, apoptosis of glial cells and to unexpected ammonium increase. Morphological changes were already observed at 2-MCA concentrations as low as 0.01 mM. Increased apoptosis and ammonium accumulation started at 0.1 mM thus suggesting that ammonium accumulation is secondary to cell suffering and/or cell death. Local accumulation of ammonium in CNS, that may remain undetected in plasma and urine, may therefore play a key role in the neuropathogenesis of methylmalonic aciduria both during acute decompensations and in chronic phases. If confirmed *in vivo*, this finding might shift the current paradigm and result in novel therapeutic strategies.

## Background

Methylmalonic acidurias (MMAurias) are a genetically heterogeneous group of inborn errors of metabolism characterized by the excretion of methylmalonic acid (MMA) in urine. Besides MMA, propionic acid (PA) and 2-methylcitric acid (2-MCA) accumulate in tissues and body fluids. Isolated MMAuria is caused by defects of the mitochondrial enzyme methylmalonyl-CoA mutase (MCM, EC 5.4.99.2) or in the synthesis of its cofactor 5’-deoxyadenosylcobalamin (AdoCbl). MCM is a key enzyme of the catabolic pathway of the amino acids L-isoleucine, L-valine, L-methionine and L-threonine, as well as of odd-chain fatty acids and the side chain of cholesterol [1]. MCM is encoded by the *MUT* gene (MIM *609058). Genetic mutations of the *MUT* gene cause MMAuria (MIM #251000) with either partial (*mut*^*-*^) or complete (*mut*^*0*^) enzyme deficiency [2]. The *cblA* type of MMAuria due to a defect in AdoCbl synthesis is caused by mutations in the *MMAA* gene encoding a protein of unknown function (MIM *607481) [3] and the *cblB* variant by mutations in the *MMAB* gene encoding cobalamin adenosyltransferase (MIM *607568) [4]. *CblA* and *cblB* variants are responsive to cobalamin supplementation [3,4].

The age of onset is variable. Most of the patients present with an acute life-threatening metabolic crisis in the first years of life, which is usually precipitated by catabolic stress (e.g. intercurrent illness). The leading clinical sign during crisis is vomiting, followed by lethargy and coma, while the biochemical profile is characterized by metabolic acidosis, hyperammonemia, hyperglycinemia and hypoglycemia [5]. Late onset forms with chronic progressive disease are also known.

The therapeutic management consists of dietary treatment (low protein diet) combined with carnitine and – in responsive forms – cobalamin supplementation. Emergency treatment aims to prevent or reverse a catabolic state by means of high energy intake. It has recently been shown that low protein diet and L-carnitine significantly reduce urinary biomarkers of protein and lipid oxidative damage [6]. Retrospective studies have shown that survival and neurological outcome were unfavorable in patients with an early onset and/or not responding to cobalamin [7]. The overall survival rate has improved during the last two decades, but the long-term outcome remains unsatisfying. Quality of life of affected patients is massively impaired by neurological (extrapyramidal movement disorder, developmental delay) and renal (chronic renal failure) complications [8,9]. Despite newborn screening and pre-symptomatic treatment of MMAuria in several countries, neurological complications remain significant in affected patients.

Liver transplantation has been used to correct the enzymatic defect in MMAuria, the liver being considered the main organ expressing the propionate catabolic pathway. However, several reports of neurological deterioration after liver transplantation can be found in the literature [8,10]. It has been shown that MMA levels in CSF stay relatively high even after liver transplantation, whereas they drop dramatically in blood and urine [11]. Since the catabolic pathway of propionate metabolism is expressed in neurons of the developing and adult CNS [12], liver transplantation likely corrects the metabolic defect only in the periphery while autonomous production of potentially neurotoxic metabolites can still occur in CNS and can lead to neurologic damage.

Different pathomechanistic concepts for brain damage in MMAuria have been proposed: MMA was first considered as the main neurotoxic metabolite, whereas other studies suggested toxic effects of propionyl-CoA and 2-MCA [13,14]. MMA has structural similarities with known inhibitors of respiratory chain complex II and was thought to be a mitochondrial toxin [15]. Toxic effects of MMA on primary neuronal cultures and in rats after intrastriatal administration have been demonstrated and could be prevented by succinate, N-methyl-D-aspartate receptor antagonists and antioxidants [14]. MMA loading in cultured rat striatal neurons resulted in intracellular accumulation not only of MMA, but also of 2-MCA and malonate [14]. Thus, impairment of energy metabolism might be mediated by a synergistic inhibition of TCA cycle and mitochondrial respiratory chain by 2-MCA, MMA and propionyl-CoA [13]. A recent study on isolated rat brain mitochondria demonstrated an inhibitory effect of MMA on α-ketoglutarate dehydrogenase. Measurements of α-ketoglutarate transport and mitochondrial MMA accumulation indicated that MMA/α-ketoglutarate exchange depletes mitochondria from this substrate. The same group, however, could not find any permanent defects on mitochondrial respiration in isolated brain mitochondria after intraperitoneal injection of MMA in young rats [16]. MMA provoked oxidative damage and compromised antioxidant defenses in nerve terminals and striatum of young rats [17]. In conclusion, the mitochondrial impairment in MMAuria seems to be a combination of inhibition of specific enzymes and transporters, limitation in the availability of substrates and oxidative damage [18].

The blood–brain barrier (BBB) has a limited transport capacity for dicarboxylates [19]. It has been hypothesized that in MMAuria brain-generated dicarboxylates might be trapped in the CNS and cause neurodegeneration. A low, but specific efflux transport via organic anion transporter 1 (OAT1) and OAT3 has been shown for MMA across porcine brain capillary endothelial cells, an *in vitro* model of the BBB [20]. It was also assumed that MMA might interfere with the transport of dicarboxylates between neurons and astrocytes [21].

During the last years brain damage has become one of the major research topics in MMAuria. The understanding of the underlying neuropathological mechanisms is crucial for the development of preventive treatments. In this study we report on the differential effects that have been observed when exposing developing brain cells in 3D organotypic rat brain cell cultures to MMA, PA and 2-MCA, the main metabolites of MMAuria.

## Methods

Unfortunately, so far there is no viable knock-out mouse model for isolated MMAuria. We decided to use the 3D rat organotypic brain cell cultures in aggregates as this *in vitro* system is well established and has been proven to be a good model for the study of inborn errors of metabolism in the CNS [22,23]. In addition to the 3-dimensional structure containing all brain cell types, the advantage of this model is the possibility to study different stages of brain cell development and maturation.

### Ethics statement

This study was carried out in strict accordance to the Ethical Principles and Guidelines for Scientific Experiments on Animals of the Swiss Academy for Medical Sciences. The protocol was approved by the http://Ethics Committee for Animal Experimentation (Service de la consommation et des affaires vétérinaires, Epalinges, Switzerland; No. 1172.5). Sufficient amount of food and water for transportation and period before sacrificing of the rats was added by the commercial provider. All animals were sacrificed 48 hours after commercial delivery by decapitation with the use of a guillotine to avoid animal suffering.

### 3D rat organotypic brain cell cultures in aggregates

Pregnant Sprague–Dawley rats (Harlan; Netherlands) were sacrificed on day 15 of gestation. Fetal whole brains were extracted, pooled and mechanically dissociated. 3.6x 10^7^ cells were grown in 8 ml of a serum-free, chemically-defined medium with 25 mM glucose and maintained under constant gyratory agitation at 37°C, in an atmosphere of 10% CO_2_ and 90% humidified air to form reaggregated 3D primary brain cell cultures as previously described [22,24]. Media were replenished every three days from day-in-vitro (DIV) 5, by exchanging 5 ml of medium per culture. On the day of harvest aggregate pellets were washed three times with ice-cold phosphate-buffered saline (PBS) and either embedded for histology in cryoform (Tissue-Tek O.C.T. Compound, Sakura Finetek, Netherlands) and frozen in liquid nitrogen-cooled 2-methylbutane (Sigma-Aldrich, Germany); or directly frozen in liquid nitrogen and kept at −80°C until analysis.

### Treatment protocols

**Figure 1:**
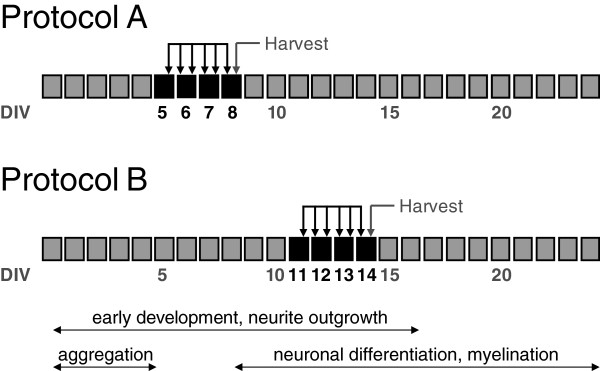
**Treatment protocols. **Brain cell aggregates were exposed to 1 mM 2-MCA, MMA and PA or lower concentrations of 2-MCA at two time points representing different developmental stages of brain cell maturation (Protocols **A **and **B**). Metabolites were added 6 times every 12 hours (indicated by arrows) starting on DIV 5 in protocol **A **and on DIV 11 in protocol **B **(treatment DIVs are indicated by black boxes) 12 hours after the change of the medium. Aggregates were harvested 5 hours after the last treatment on DIV 8 in protocol A and on DIV 14 in protocol **B**.

Cultures were treated with 0.01, 0.1, 0.33 and 1 mM 2-MCA (Ernesto Brunet-Romero, Madrid, Spain), 1 mM MMA (Fluka, No 67750, Switzerland) or 1 mM sodium propionate (PA, Sigma-Aldrich, N° P1880, Germany) buffered in 25 mM HEPES (Sigma-Aldrich, Germany) and with pH adjusted to 7.5. Cultures were exposed to one of the three metabolites 6 times every 12 hours at two different developmental stages starting from DIV 5 in protocol A or from DIV 11 in protocol B (Figure 1). Aggregates were harvested 5 hours after the last treatment at DIV 8 in protocol A and at DIV 14 in protocol B.

### Immunohistochemistry

Immunohistochemical staining was carried out on 16 μm aggregate cryosections using antibodies against different markers of brain cell types: phosphorylated medium weight neurofilament (p-NFM; clone NN18, Sigma-Aldrich, USA) for neurons [25], glial fibrillary acidic protein (GFAP; Millipore, USA) for astrocytes, galactocerebroside (GalC; Millipore, USA) on DIV 8 and myelin basic protein (MBP; Santa Cruz Biotechnology, USA) on DIV 14 for oligodendrocytes, and peroxidase-labeled isolectin B4 (Sigma-Aldrich, USA) on DIV 8 for microglia. Briefly, sections were fixed for 1 h in 4% paraformaldehyde in PBS (Sigma-Aldrich, Germany) at room temperature. Endogenous peroxidase activity was quenched with 1.5% H_2_O_2_ in PBS (Sigma-Aldrich, Germany) and non-specific antibody binding sites were blocked with 1% bovine serum albumin in PBS (BSA-PBS; Sigma-Aldrich, Germany) for 1 h. Primary antibodies diluted 1:100 in 1% BSA-PBS where applied to sections and further detected with anti-mouse or anti-rabbit IgG coupled to horseradish peroxidase (HRP, Bio-Rad Laboratories, USA). Staining was processed using the AEC Substrate Set for BD™ ELISPOT according to the manufacturer's protocol (BD Biosciences, USA). For negative controls, primary antibodies were omitted resulting in no staining. The stained sections were mounted under FluorSave™ Reagent (Calbiochem, USA), observed and digitized using an Olympus BX50 microscope equipped with a UC30 digital camera (Olympus, Japan).

### Immunofluorescence

Detection of cleaved caspase-3 in aggregates was performed with the Tyramide Signal Amplification Kit (Life Technologies, USA). Aggregate cryosections (16 μm) were subjected to the same procedure as described above for immunohistochemistry. Non-specific antibody binding sites were blocked for 1 h at room temperature with the blocking buffer of the kit. The primary antibody against the large fragment (17/19kDa) of activated caspase-3 (Cell Signaling Technology, USA) diluted 1:1000 in blocking buffer was applied to sections overnight at 4°C. After washing, sections were incubated with a HRP anti-rabbit IgG secondary antibody (provided by the kit) for 1 h. Peroxidase staining was performed using Alexa Fluor® 555-labeled tyramide diluted 1:200 in amplification buffer (provided by the kit) and applied to sections for 10 min. Negative controls were processed the same but omitting the primary antibody, resulting in no staining. Sections were mounted under FluorSave™ reagent. The sections were observed and photographed with an Olympus BX50 microscope equipped with a UC30 digital camera.

### *In situ* cell death detection

To detect typical features of apoptosis (fragmented nuclei, apoptotic bodies), nuclear DNA was stained using the blue fluorescent 4',6-diamidino-2-phenylindole (DAPI, Invitrogen, USA). Aggregate cryosections (16 μm) were incubated with DAPI for 10 min at room temperature. *In situ* detection of cell death was performed using terminal deoxynucleotidyl transferase (TdT)-mediated dUTP nick end labeling (TUNEL) on 16 μm cryosections of aggregates. TUNEL staining was performed according to supplier recommendations using the *In Situ* Cell Death Detection Kit with Fluorescein (Roche Applied Science, Switzerland) resulting in green fluorescence in dying cells.

### Western blot analysis

Aggregates were homogenized in 150 mM sodium chloride, 50 mM Tris–HCl, pH 8.0, 1% NP-40 (Sigma-Aldrich, Germany) and Protease Inhibitor Cocktail - Complete Mini (Roche Applied Science, Switzerland) and sonicated for 5 seconds. Lysates were cleared by centrifugation at 12’000 rpm for 30 min at 4°C. After dilution, protein content was measured by bicinchoninic acid assay (BCA) (Thermo Scientific, USA) and diluted to a final concentration of 1.2 μg/μl in NuPAGE® LDS Sample Buffer (Life Technologies, USA). Samples were heated at 70°C for 10 min and resolved on NuPAGE® 4-12% Bis–Tris Gel (for p-NFM) or NuPAGE® 12% Bis–Tris Gel (for GFAP, MBP, actin and caspase-3) using NuPAGE® MOPS SDS Running Buffer (Life Technologies, USA) at a constant voltage (200 V, 60 min). Proteins were transferred onto Immobilon-FL PVDF, 0.45 μm membranes (Millipore, USA). Membranes were blocked with 5% non-fat dry milk in TBS-Tween (20 mM Trizma base, 137 mM NaCl, 0.05% Tween, pH 7.6) for 1 h at room temperature. After blocking, the membranes were incubated overnight with different primary antibodies against GFAP, MBP, p-NFM, Actin (I-19) (Santa Cruz Biotechnology, USA) or full-length (35kDa) and large fragment (17/19 kDa) of caspase-3 (Cell Signaling Technology, USA) diluted 1:1000 in 3% dry milk and TBS-Tween. The membranes were probed with HRP-conjugated goat anti-mouse IgG or goat anti-rabbit IgG (1:3000; Vector laboratories, USA) and developed by chemiluminescence (ECL Western Blotting Detection Reagents; GE Healthcare, France). Blots were stripped (ReBlot Plus Mild Antibody Stripping Solution; Millipore, USA) and re-probed with antibody against actin as the loading control. Images were taken with a Luminescent Image Analyzer LAS-4000 (Fujifilm; Life Science) and quantified with the public Java-based image processing program ImageJ (National Institutes of Health). Data acquired in arbitrary densitometric units were normalized to actin and transformed to percentages of the densitometric levels obtained from scans of control samples visualized on the same blots.

### Measure of basic metabolites and amino acids in the culture media

Ammonium was measured on an Integra automatic analyzer (Roche); glucose, lactate and lactate dehydrogenase (LDH) were measured on a Modular automatic analyzer (Roche); free amino acids were analyzed on a Beckman 6300 amino acid analyzer; as described previously [26].

### Statistics

All data points are expressed as mean ± standard error of the mean (SEM). Statistical difference was determined with Student’s *t*-test.

## Results and discussion

We used 3D organotypic brain cell cultures in aggregates to explore, *in vitro*, the effects of the three main metabolites (2-MCA, MMA and PA) accumulated in body fluids of subjects affected by MMAuria. Our *in vitro* model is particularly suitable for studying neurotoxicity because the aggregates contain all types of brain cells with their spontaneous connections between each other. In addition, this model reproduces early phases of brain development and has proven to be optimal to study differential effects of metabolic derangements (such as hyperammonemia) in developing brain compared to adult brain tissue [22-25]. This is particularly important when studying a disorder in which the most dramatic brain damage occurs in early childhood.

### Morphological changes of developing brain cells in our *in vitro* model for MMAuria

#### Neurons

**Figure 2:**
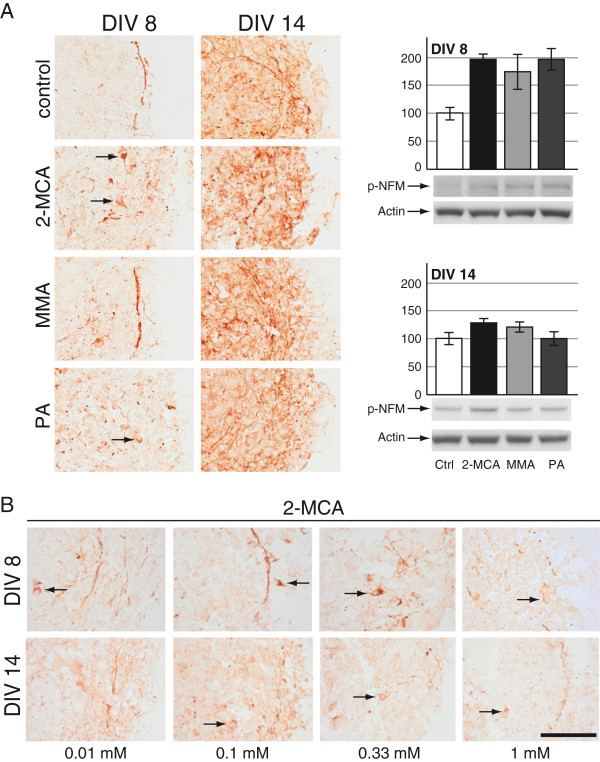
**Effects of 2-MCA, MMA and PA on neurons. **Immunohistochemical staining for phosphorylated medium weight neurofilament (p-NFM) on cryosections of cultures derived from DIV 8 and DIV 14. Cultures were exposed to 1 mM 2-MCA, MMA or PA (**A**; left panel) or lower concentrations of 2-MCA (**B**). Stained cell bodies are indicated by black arrows. Scale bar: 100 μm. **A**; right panel: Representative western blots with data quantification of whole-cell lysates for p-NFM on DIV 8 (above) and on DIV 14 (below). Actin was used as a loading control. The quantifications of p-NFM normalized to actin are expressed as percentage of respective controls. The values represent the mean ± SEM from three replicates taken from two independent experiments.

Immunohistochemical staining for p-NFM that normally only labels axons showed a substantial decrease of axonal labeling at DIV 8, and interestingly a retention of the signal in neuronal bodies at DIV 8 and 14, after exposure to 2-MCA and to a lesser extend to PA on DIV 8 (Figure 2A; left panel). For 2-MCA, signal retention was already observed at 0.01 mM, the lowest tested concentration, on DIV 8 and at 0.1 mM on DIV 14 (Figure 2B). In contrast, MMA exposure on DIV 8 induced a slight increase of p-NFM expression, without altering neuronal and axonal morphology (Figure 2A; left panel, DIV 8). This signal increase may be explained by a stimulatory effect of MMA on neuronal growth or differentiation, as also described below for oligodendrocytes. The semi-quantitative analysis of p-NFM expression by western blotting showed an increase in cultures treated with all metabolites compared to control on DIV 8 (Figure 2A; right panel). Higher p-NFM expression after 2-MCA and PA exposure on DIV 8 is most likely an artifact which can probably be explained by the deleterious effect on the overall cell survival leading to diminished total protein content in these cultures. Since western blots are performed with a defined protein quantity this might lead to the appearance of a falsely increased expression of this protein.

#### Astrocytes

**Figure 3:**
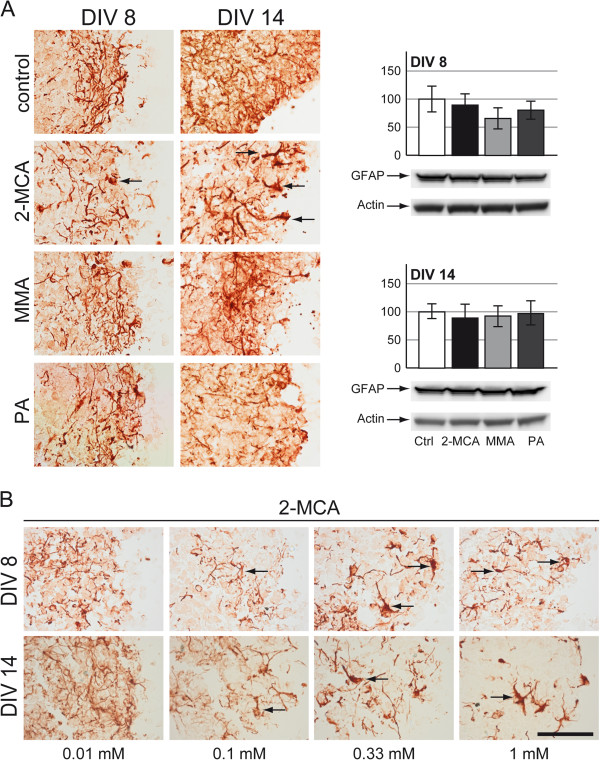
**Effects of 2-MCA, MMA and PA on astrocytes. **Immunohistochemical staining for glial fibrillary acidic protein (GFAP) on cryosections of cultures on DIV 8 and 14. Cultures were exposed to 1 mM 2-MCA, MMA or PA (**A**; left panel) or lower concentrations of 2-MCA (**B**). Swollen proximal fibers are indicated by black arrows. Scale bar: 100 μm. **A**; right panel: Representative western blots with data quantification of whole-cell lysates for GFAP on DIV 8 (above) and on DIV 14 (below). Actin was used as a loading control. The quantifications of GFAP levels normalized to actin are expressed as percentage of respective controls. The values represent the mean ± SEM from three replicates taken from two independent experiments.

Compared to controls aggregates treated with 2-MCA showed a significant decrease in astrocyte fiber density, an enlargement of astrocytic bodies and a swelling of proximal fibers (Figure 3A, left panel). On DIV 8 a decrease of fiber density was already observed after exposure to 0.01 mM 2-MCA, followed by the appearance of swollen fibers and astrocytic bodies at 0.1 mM. On DIV 14 enlarged astrocytic bodies were already observed at 0.01 mM, whereas a decreased fiber density and swollen fibers were only present from concentrations of 0.1 mM on (Figure 3B). We speculate that this is the early appearance of suffering astrocytes that are going to develop a major swelling. Exposure to PA on DIV 14 revealed a slight decrease in the density of astrocytic fibers (Figure 3A; left panel). Aggregates treated with PA (DIV 8) or with MMA (DIV 8 and 14) did not show any significant changes compared to controls (Figure 3A; left panel). A western blot quantification of GFAP expression was performed and did not present any significant alteration for any of the treatments (Figure 3A; right panel).

#### Oligodendrocytes

**Figure 4:**
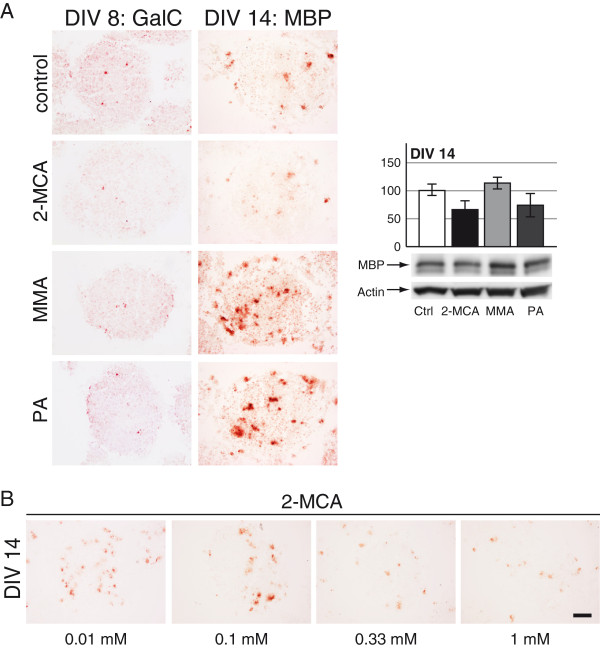
**Effects of 2-MCA, MMA and PA on oligodendrocytes. **Immunohistochemical staining for galactocerebroside (GalC, DIV 8) and myelin basic protein (MBP, DIV 14) on cryosections of cultures on DIV 8 and 14. Cultures were exposed to 1 mM 2-MCA, MMA or PA (**A**; left panel) or lower concentrations of 2-MCA (**B**). Scale bar: 100 μm. **A**; right panel: Representative western blots with data quantification of whole-cell lysates for MBP on DIV 14. Actin was used as a loading control. The quantifications of MBP levels normalized to actin are expressed as percentage of respective controls. The values represent the mean ± SEM from three replicates taken from two independent experiments.

Oligodendrocytes in immature cultures (DIV 8) were studied with GalC, which is one of the earliest markers for this cell type. We did not observe any substantial effect on GalC signal for the treatments on DIV 8 (Figure 4A; left panel; DIV 8). For more developed cultures (DIV 14) MBP staining was used. Treatment with 2-MCA on DIV 14 revealed a signal loss for MBP, whereas exposure to MMA and PA resulted in a significant signal increase (Figure 4A; left panel; DIV 14). In the dose–response experiment for 2-MCA a slight decrease of MBP signal was already observed at 0.1 mM (Figure 4B). Western blotting of cultures from DIV 14 confirmed a slightly increased expression of MBP after MMA treatment and the decreased MBP expression after 2-MCA exposure (Figure 4A; right panel).

#### Microglia

The presence of microglia was tested by immunostaining for isolectin on DIV 8. No interesting changes were observed (data not shown).

In contrast to previous studies [14] we could not show any deleterious effects of MMA on brain cell morphology in our *in vitro* model for MMAuria. The only observation that differed from controls was a slight stimulation of neuronal and oligodendrocytic growth or differentiation on DIV 8 and DIV 14, respectively. This finding and its consequences for the developing brain has to be further assessed. 2-MCA turned out to be the most destructive metabolite in our model with effects on cell morphology already visible at concentrations as low as 0.01 mM. Light microscopy showed that astrocytes at both developmental stages severely suffered from 2-MCA treatment showing fiber reduction and proximal fiber swelling. Further, 2-MCA exposure of immature cultures (DIV 8) resulted in diminished axonal outgrowth, retarded neuronal differentiation or axonal degeneration, also observed on DIV 8 and DIV 14 by p-NFM accumulation in neuronal cell bodies. In more developed cultures (DIV 14) 2-MCA exposure let to retarded differentiation of oligodendrocytes. PA exposure had less pronounced effects on neuronal differentiation on DIV 8 and subtle oligodendrocytic boosting on DIV 14.

### Biochemical changes in culture media in our *in vitro* model for MMAuria

#### Glucose and Lactate

**Figure 5:**
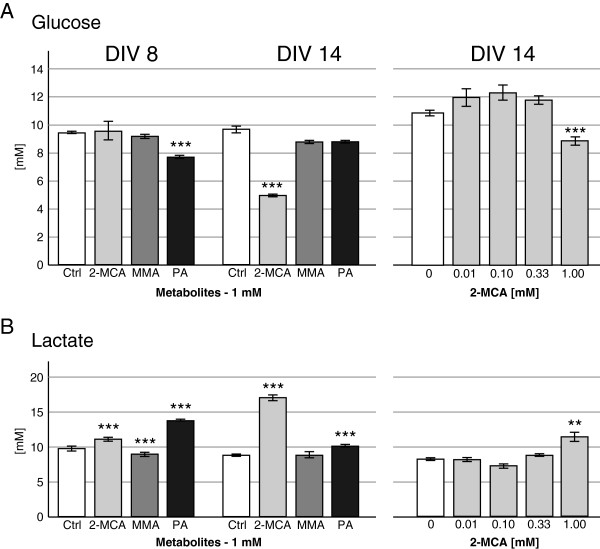
**Effects of 2-MCA, MMA and PA on glucose and lactate levels. **Glucose (**A**) and lactate (**B**) were measured in the medium of cultures from DIV 8 and DIV 14. Cultures were exposed to 1 mM 2-MCA, MMA or PA (**A** and **B**; left panel, DIV 8 and 14) or lower concentrations of 2-MCA (**A** and **B**; right panel, DIV 14). Mean ± SEM of 4 to 7 replicate cultures assessed by Student’s *t*-test; **p<0.01, *** p<0.001.

As compared to controls, PA exposure on DIV 8 and 2-MCA exposure on DIV 14 caused a significant decrease in the glucose levels, while glucose levels of cultures exposed to MMA did not change significantly (Figure 5A). In parallel to decreased glucose levels, a significant increase in lactate levels was measured in the medium of cultures treated with 2-MCA and PA on DIV 8 and 14 (Figure 5B). No significant changes of glucose or lactate levels were observed for 2-MCA concentrations below 1 mM on DIV 8 (data not shown) and 14 (Figure 5A and B, right panel).

#### Ammonium and Glutamine

**Figure 6:**
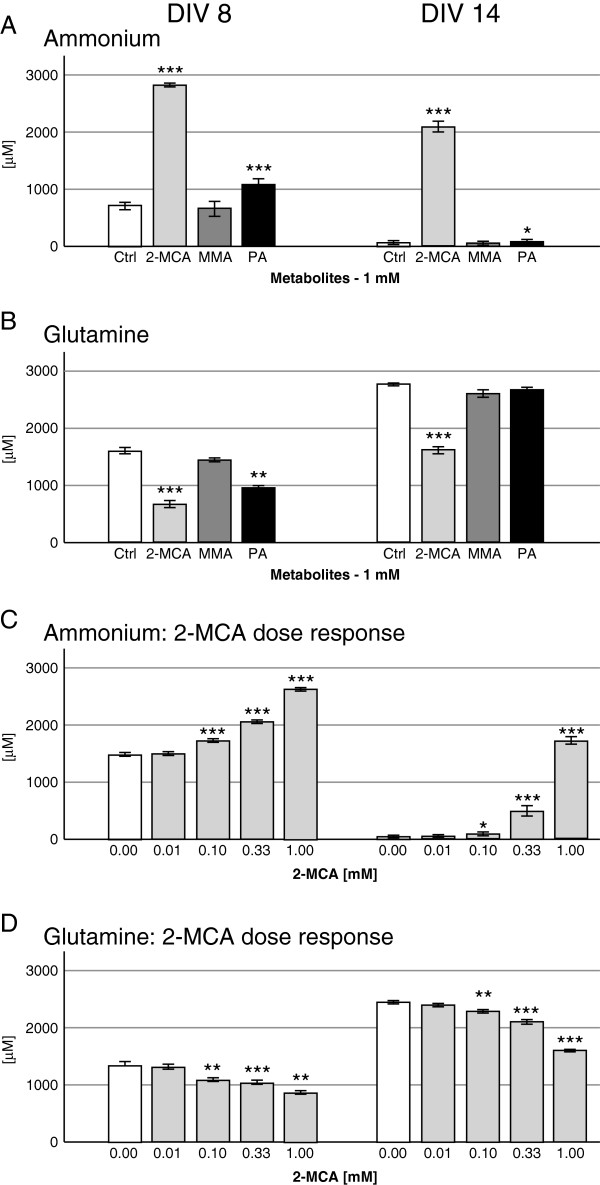
**Effects of 2-MCA, MMA and PA on ammonium and glutamine levels.** Ammonium (**A **and **C**) and glutamine (**B **and **D**) were measured in the medium of cultures from DIV 8 and DIV 14. Cultures were exposed to 1 mM 2-MCA, MMA or PA (**A **and **B**) or lower concentrations of 2-MCA (**C **and **D**). Mean ± SEM of 4 to 7 replicate cultures assessed by Student’s *t*-test; *p<0.05, **p<0.01, *** p<0.001.

A significant increase in ammonium concentrations was measured in culture media after exposure to 2-MCA and PA on DIV 8 and 14 with a 25-fold ammonium increase after 2-MCA treatment on DIV 14 (Figure 6A). In parallel to the described increase of ammonium levels a significant decrease of glutamine in the culture media of cultures treated with 2-MCA on DIV 8 and 14 and to a lesser extent with PA on DIV 8 was observed (Figure 6B). Dose–response experiments with lower 2-MCA concentrations revealed a significant change of ammonium and glutamine starting at 0.1 mM (Figure 6C and D).

For human hyperammonemic patients, data are lacking on extracellular brain ammonium concentration. However, serum levels of ammonium leading to irreversible damage to the developing CNS can peak as high as 2000 μM, usually after chronic hyperammonemia in the range of 200 μM [27]. The correlation of hyperammonemia and decreased glutamine levels has already been observed in patients with propionic acidemia. The authors discussed a potential inhibition of the enzyme glutamine synthetase, but conclude due to a series of other metabolic alterations observed in patients that hyperammonemia can more likely be explained by the inability to maintain adequate levels of glutamine precursors through a dysfunctional Krebs cycle [28]. The observed ammonia concentrations are comparable to those applied to the same *in vitro* model to induce brain cell damage when modeling urea cycle disorders [25].

The biochemical parameters confirm the observations from brain cell morphology concerning the toxicity of the three metabolites: MMA exposure does not significantly alter the metabolism of brain cells. Surprisingly, media of cultures treated with 2-MCA contained 25-fold more ammonium than controls. Ammonium is a well known toxin for the CNS. It has been shown to have an influence on axonal elongation [25], to provoke astrocytic swelling and to contribute to white matter changes observed in patients suffering from primary hyperammonemia [29]. This could implicate that patients with MMAuria during crisis might suffer from a more severe intracerebral ammonium accumulation than predicted by blood hyperammonemia and may also suffer from cerebral ammonia intoxication during chronic illness, leading to brain damage even in the absence of metabolic acidosis and hyperammonemia. If confirmed by further investigations *in vivo*, this finding might open new therapeutic perspectives. The cause of ammonium accumulation in brain cells is unclear. Urea cycle enzymes are incompletely expressed in brain; amino acid catabolism in brain cells produces ammonium at a slower rate than in liver and therefore glutamine synthesis is sufficient to avoid ammonia accumulation. The astrocytic ATP-dependent enzyme glutamine synthetase acts under physiological conditions as a buffering system for detoxification of ammonium in the CNS. In conditions of elevated ammonium, glutamine is expected to increase accordingly. Surprisingly, media of 2-MCA-treated cultures revealed, besides elevated ammonium, significantly decreased glutamine levels. This suggests that ammonium accumulation is the consequence of either 2-MCA related inhibition of glutamine synthetase activity, astrocytic death leading to lack of glutamine synthetase, or energy failure with lack of ATP resulting in inhibition of glutamine synthetase activity.

2-MCA treatment further resulted in a decrease of glucose levels and an increase of lactate in culture media. Low concentration studies for 2-MCA exposure revealed that this effect cannot be observed after exposure to less than 1 mM suggesting that this is a secondary effect in the neuropathogenesis. The combination of decreased glucose levels and increased lactate production is also observed in patients’ blood during metabolic crisis. Underlying mechanisms may be the uncoupling of the respiratory chain as suggested by previous publications [18] and/or the inhibition of gluconeogenesis. In *Salmonella enterica*, 2-MCA was found to inhibit fructose-1,6-biphosphatase, the key enzyme for gluconeogenesis, and to interfere with cell growth. This effect was overcome by increase of glucose concentration in the culture medium [30]. Culture exposure to PA reveals similar, but less impressive effects on biochemical parameters in culture media in comparison to 2-MCA treatment. As biochemical changes are only observed with higher 2-MCA concentrations than the morphological changes they might not be the origin but the consequence of 2-MCA toxicity on the different brain cell types.

### 2-MCA induced apoptosis in developing brain cells

**Figure 7:**
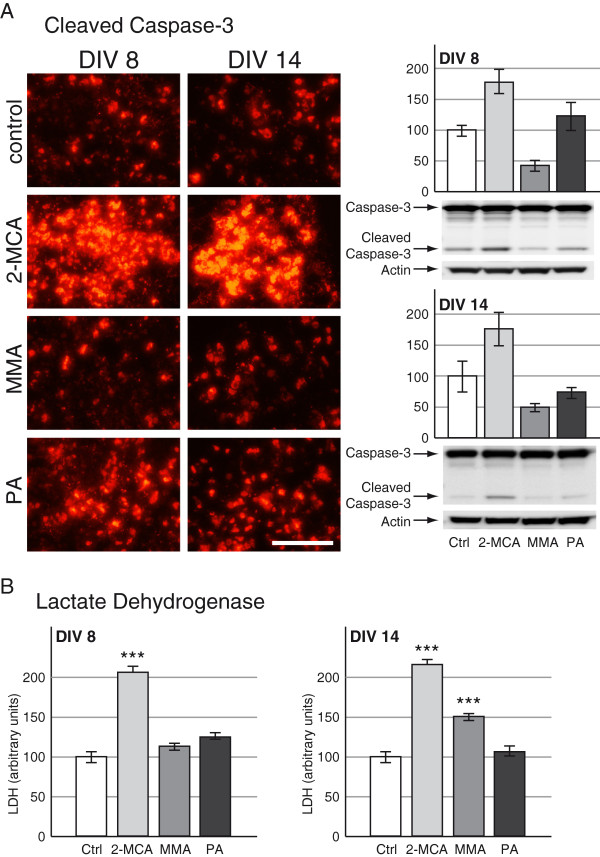
**Evaluation of cell death after exposure to 2-MCA, MMA and PA. A**; left panel: Immunohistochemical staining for cleaved caspase-3 (red signal). Scale bar: 100 μm. **A**; right panel: Representative western blots with data quantification of whole-cell lysates for full length caspase-3 and the large fragment of cleaved caspase-3 (i.e. activated caspase-3) on DIV 8 (left) and on DIV 14 (right). Actin was used as a loading control. The quantifications of cleaved caspase-3 normalized to actin are expressed as percentage of respective controls. The values represent the mean ± SEM from three replicates taken from two independent experiments. **B**: LDH in culture medium of cultures from DIV 8 (left) and DIV 14 (right). Mean ± SEM of seven replicate cultures assessed by Student’s *t*-test; *** p<0.001.

**Figure 8:**
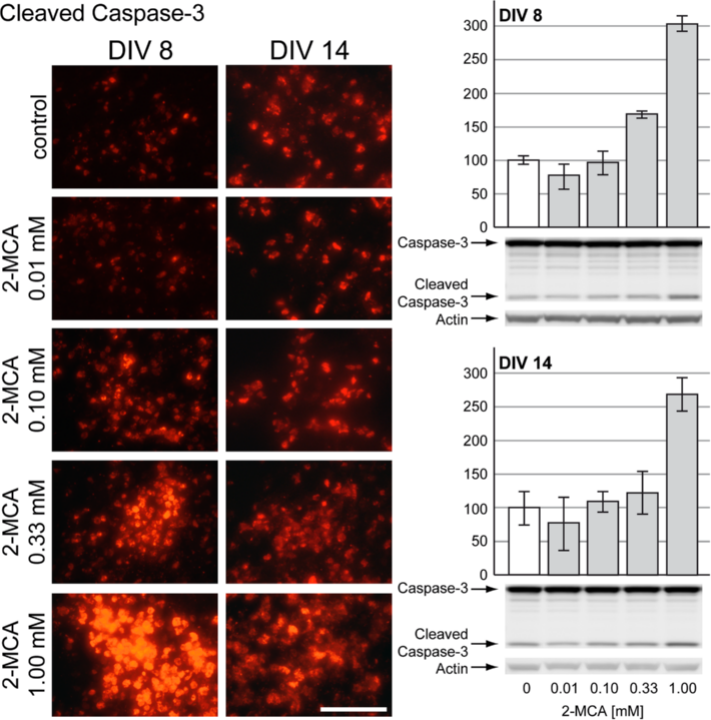
**Evaluation of cell death after exposure to with lower concentrations of 2-MCA.** Left panel: Immunohistochemical staining for cleaved caspase-3 (red signal). Scale bar: 100 μm. Right panel: Representative western blots with data quantification of whole-cell lysates for full length caspase-3 and the large fragment of cleaved caspase-3 (i.e. activated caspase-3) for DIV 8 (above) and DIV 14 (below). Actin was used as a loading control. The quantifications of cleaved caspase-3 normalized to actin are expressed as percentage of respective controls. The values represent the mean ± SEM from three replicates.

Lactate dehydrogenase (LDH) was measured in culture medium and was substantially increased after 2-MCA exposure on DIV 8 and after 2-MCA or MMA exposure on DIV 14 (Figure 7B). This observation indicated a possible increase of cell death in these cultures. To evaluate cell death, we performed TUNEL, DAPI and activated caspase-3 immunofluorescence labeling. DAPI staining showed an increased appearance of nuclear fragmentation and apoptotic bodies in cultures treated with 2-MCA and PA in both protocols (data not shown). Staining for TUNEL showed an important signal increase in cultures treated with 2-MCA on DIV 8 and 14 (data not shown). These findings were confirmed by the observation of a massively increased number of apoptotic cells in 2-MCA-treated cultures and a less pronounced increase of apoptotic cells in PA-treated cultures derived from both protocols (DIV 8 and 14) after staining for activated caspase-3 (Figure 7A; left panel). Increased activation of caspase-3 after treatment with 2-MCA on DIV 8 and DIV 14 or PA on DIV 8 was also confirmed by western blots (Figure 7A; right panel). Interestingly, a substantial decrease in the apoptosis rate was observed in MMA-exposed cells in both protocols (Figure 7A; left panel). Dose–response experiments with lower 2-MCA concentrations showed an increase of cleaved caspase-3 signal starting from 0.1 mM on DIV 8 and from 0.33 mM on DIV 14 (Figure 8, left panel). Western blots for cleaved caspase-3 revealed a substantially increased activation starting at 0.33 mM on DIV 8 and at 1 mM on DIV 14 (Figure 8, right panel). On DIV 14, we observed an increased apoptosis rate only at higher concentrations of 2-MCA (0.33 and 1 mM) compared to DIV 8, where we see the effect already at 0.1 mM 2-MCA. This is probably due to an advanced maturation of the cells in our aggregates.

Consistent with our findings on cell morphology and metabolism, 2-MCA appears to be the most toxic metabolite in our *in vitro* model leading to massive apoptosis. Unfortunately, there is no data in the literature about intracerebral concentrations of 2-MCA in MMAuria patients. Given the range of 2-MCA concentrations found in plasma, urine or CSF of patients, the observed effect of altered cell morphology starting already at 0.01 mM, and of increased apoptosis starting at 0.1 mM might however reflect the *in vivo* situation.

PA also has some destructive effects on cell survival that are less striking than those of 2-MCA. The significant decrease of apoptosis rate after MMA exposure remains unexplained and needs to be further investigated.

The dying cells are most likely glial cells that could be more sensitive to 2-MCA as they do not express the enzymes of the catabolic propionate pathway [12]. Cell death is also a known toxic effect of ammonium in the CNS if exposed to high concentrations (5 mM) or prolonged in time [31]. There is striking parallelism between increase of ammonium, decrease of glutamine and increase of apoptosis starting at 0.1 mM 2-MCA on DIV 8 and at 0.33 mM on DIV 14 which supports the hypothesis that there is a direct link between the observed effects.

In our study we showed that 2-MCA is the most toxic metabolite for brain cells under development. This is of particular interest as 2-MCA is also a key metabolite in another organic aciduria, namely propionic aciduria, where it reaches comparable or even higher levels in body fluids of patients. Patients suffering from propionic aciduria show neurological symptoms similar to those observed in MMAuria [32]. It is thus likely, that the same mechanisms contribute to neuropathophysiology in both diseases.

## Conclusions

In our model of 3D rat organotypic brain cell cultures in aggregates, low concentrations of 2-MCA already have deleterious effects on neurons (limited axonal growth) and glial cells (cell swelling and massive apoptosis). These effects seem to precede a striking ammonium accumulation and increased apoptosis. These observations indicate that ammonium may be a key player in the neuropathogenesis of MMAuria and therefore might be a potential target for neuroprotective measures. Ammonium accumulation might be responsible for brain damage in MMAuria patients not only during metabolic crises but also on chronic disease course and even after liver transplantation.

## Abbreviations

2-MCA: 2-methylcitrate; AdoCbl: 5-deoxyadenosylcobalamin; BBB: Blood–brain barrier; BCA: Bicinchoninic acid; BSA: Bovine serum albumin; CNS: Central nervous system; CSF: Cerebrospinal fluid; DAPI: 4',6-diamidino-2-phenylindole; DIV: Day-in-vitro; GalC: Galactocerebroside; GFAP: Glial fibrillary acidic protein; HRP: Horse radish peroxidase; LDH: Lactate dehydrogenase; MBP: Myelin basic protein; MCM: Methylmalonyl-CoA mutase; MMA: Methylmalonic acid; methylmalonate; MMAuria: Methylmalonic aciduria; OAT: Organic anion transporter; PA: Propionic acid; propionate; PBS: Phosphate-buffered saline; PFA: Paraformaldehyde; p-NFM: Phosphorylated medium weight neurofilament; TCA cycle: Tricarboxylic acid cycle; TUNEL: Terminal deoxynucleotidyl transferase (TdT)-mediated dUTP nick end labeling.

## Competing interests

The authors declare no competing interests.

## Authors’ contributions

DB determined the experimental design, established the *in vitro* model for MMAuria, participated on experimental procedures, supervised the work of PJ and PZ and wrote the manuscript. PJ was in charge of the cultures, did most of the immunohistochemistry and the western blots. OB participated in experimental procedures, in the supervision of PJ and PZ particularly with his great experience in the 3D model and in microscopy and contributed to manuscript writing. PZ did the immunofluorescence assays for apoptosis and participated in the cultures. HH performed the biochemical analyses in the culture media. LB contributed to data interpretation and revised the manuscript. All authors read and approved the final manuscript.
